# Emergence and spread of a SARS-CoV-2 lineage A variant (A.23.1) with altered spike protein in Uganda

**DOI:** 10.1038/s41564-021-00933-9

**Published:** 2021-06-23

**Authors:** Daniel Lule Bugembe, My V. T. Phan, Isaac Ssewanyana, Patrick Semanda, Hellen Nansumba, Beatrice Dhaala, Susan Nabadda, Áine Niamh O’Toole, Andrew Rambaut, Pontiano Kaleebu, Matthew Cotten

**Affiliations:** 1 Medical Research Council/Uganda Virus Research Institute, London School of Hygiene & Tropical Medicine Uganda Research Unit, Entebbe, Uganda; 2 Central Public Health Laboratories of the Republic of Uganda, Kampala, Uganda; 3 Institute for Evolutionary Biology, University of Edinburgh, Edinburgh, UK; 4 Uganda Virus Research Institute, Entebbe, Uganda; 5 Medical Research Council-University of Glasgow Centre for Virus Research, Glasgow, UK

**Keywords:** Viral infection, SARS-CoV-2

## Abstract

Here, we report SARS-CoV-2 genomic surveillance from March 2020 until January 2021 in Uganda, a landlocked East African country with a population of approximately 40 million people. We report 322 full SARS-CoV-2 genomes from 39,424 reported SARS-CoV-2 infections, thus representing 0.8% of the reported cases. Phylogenetic analyses of these sequences revealed the emergence of lineage A.23.1 from lineage A.23. Lineage A.23.1 represented 88% of the genomes observed in December 2020, then 100% of the genomes observed in January 2021. The A.23.1 lineage was also reported in 26 other countries. Although the precise changes in A.23.1 differ from those reported in the first three SARS-CoV-2 variants of concern (VOCs), the A.23.1 spike-protein-coding region has changes similar to VOCs including a change at position 613, a change in the furin cleavage site that extends the basic amino acid motif and multiple changes in the immunogenic N-terminal domain. In addition, the A.23.1 lineage has changes in non-spike proteins including nsp6, ORF8 and ORF9 that are also altered in other VOCs. The clinical impact of the A.23.1 variant is not yet clear and it has not been designated as a VOC. However, our findings of emergence and spread of this variant indicate that careful monitoring of this variant, together with assessment of the consequences of the spike protein changes for COVID-19 vaccine performance, are advisable.

## Abstract (web summary)

The SARS-CoV-2 variant A.23.1 became predominant in Uganda and shows amino acid changes within the S protein and non-spike proteins similar to known variants of concern.

## Main

The new severe acute respiratory syndrome coronavirus 2 (SARS-CoV-2)^1^ and the associated coronavirus disease 2019 (COVID-19)^2,3^ continue to spread throughout the world, causing >120 million infections and >2.6 million deaths (16 March 2021, Johns Hopkins COVID-19 Dashboard). Genomic surveillance has played a key role in the response to the pandemic; sequenced data from SARS-CoV-2 provides information on the transmission patterns and evolution of the virus as it enters new regions and spreads. As COVID-19 vaccines become available and are implemented, monitoring SARS-CoV-2 genetic changes, especially changes at the epitopes with implications for immune escape is crucial. A detailed classification system has been defined to help monitor SARS-CoV-2 as it evolves^4^, with virus sequences classified into two main phylogenetic lineages (Pango lineages) A and B, representing the earliest divergence of SARS-CoV-2 in the pandemic and then into sublineages within these. Several variants of concern (VOCs) have emerged showing increased transmission patterns and reduced susceptibility to vaccine and/or therapeutic antibody treatments. These VOCs include lineage B.1.1.7, first identified in the UK^5^, B.1.351 in South Africa^6^ and lineage P.1 (B.1.1.28.1) in Brazil^7^.

## Status of the SARS-CoV-2 epidemic in Uganda

SARS-CoV-2 infection was first detected in Uganda in March 2020, initially among international travellers until passenger flights were stopped in late March 2020. A second route of virus entry with truck drivers from adjacent countries then became apparent^8^. Since August 2020, community transmission dominated the Uganda case numbers. By March 2021, total cases in Uganda were 40,535, with 334 deaths attributed to the virus. We have continued our efforts to generate SARS-CoV-2 genomic sequence data to monitor virus movement and genetic changes and we report in this article on a new sublineage A (A.23.1) that has emerged and is dominating the local epidemic. The A.23.1 variant encodes multiple changes in the spike protein as well as in nsp6, ORF8 and ORF9, some predicted to be functionally similar to those observed in VOCs in lineage B.

## Changes in prevalence of lineage A viruses

**Fig. 1:**
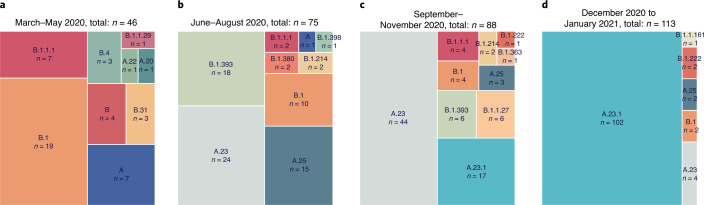
SARS-CoV-2 lineage diversity in Uganda. All high-coverage complete sequences from Uganda (*n* = 322) were lineage-typed using the pangolin resource (https://github.com/cov-lineages/pangolin). Lineage counts were stratified into four periods: March–May 2020 (**a**); June–August 2020 (**b**); September–November 2020 (**c**); and December 2020 to January 2021 (**d**). The percentage of each lineage within each set was plotted as a treemap using squarified treemap^41^ implemented in squarify (https://github.com/laserson/squarify) with the size of each sector proportional to the number of genomes; genome numbers are listed with ‘*n* = ’. Source data

The genomes generated in this study were classified into Pango lineages^4^ using the pangolin module pangoLEARN (https://github.com/cov-lineages/pangolin) and into Nextstrain clades using Nextclade^9^ (https://clades.nextstrain.org/). The distribution of virus lineages circulating in Uganda changed dramatically over the course of the year. A clear feature of the earlier COVID-19 epidemic in the country was the diversity of viruses found throughout the country attributed to frequent flights into Uganda from Europe, UK, US and Asia; this is reflected in the nine lineages seen from March to May 2020 with a mixture of both lineage A and B viruses (Fig. 1a). After passenger flights were limited in March 2020, the virus entered by land travel via truck drivers. Uganda is landlocked country, characterized by its important geographical position, that is, the crossing of two main routes of the Trans-Africa Highway in East Africa. The essential nature of produce and goods transport allowed virus movement from/to Kenya, South Sudan, Democratic Republic of the Congo, Rwanda and Tanzania. In the period from June to August 2020, the lineage B.1 and B.1.393 strains were abundant, similar to patterns observed in Kenya^10^ (Fig. 1b) although lineage A viruses did not decline as seen in US and Europe. Lineage A.23 strains were first observed in two prison outbreaks in Amuru and Kitgum, Uganda in August 2020; by September–November, A.23 was the major lineage circulating throughout the country (Fig. 1c). The A.23 virus continued to evolve into the A.23.1 lineage, first observed in late October 2020. Given the diversity of virus lineages found in the country from March until November 2020, it was unexpected that by late December 2020 to January 2021, lineage A.23.1 viruses represented 90% (102 of 113 genomes) of all viruses observed in Uganda (Fig. 1d). In all time periods, the SARS-CoV-2-positive sample were obtained from multiple clinical and surveillance locations throughout Uganda (Extended Data Fig. 5b), indicating that the differences are unlikely to be due to sampling different subpopulations in the country at different times.

## Virus sequence diversity

**Fig. 2:**
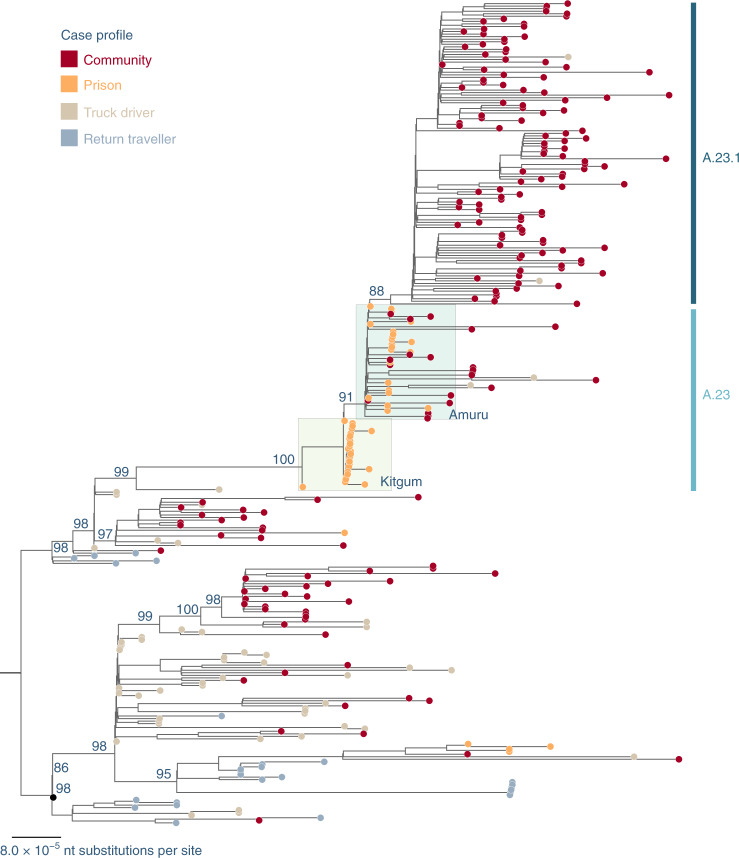
Maximum-likelihood phylogenetic tree comparing all available complete and high-coverage Uganda sequences (*n* = 322). Strain names are coloured according to the case profile: cases from the community, dark red; prison, orange; truck driver, light brown; return traveller, light blue. The case clusters from prisons in Kitgum and Amuru are highlighted in colour boxes in light yellow and light green, respectively. Lineages A.23 and A.23.1 are indicated. The tree was rooted where lineages A and B were split. Branch length is drawn to the scale of the number of nucleotide substitutions per site, indicated in the lower left; only bootstrap values of major nodes are shown. Source data

All newly and previously generated Uganda genomes that were complete and high-coverage (*n* = 322) were used to construct a maximum-likelihood phylogenetic tree (Fig. 2).

A number of A and B variant lineages were observed briefly at low frequencies and may have undergone extinction, similar to patterns observed in the UK^11,12^. Although based on limited sampling, genomes identified from a truck driver are often observed basal to community clusters (Fig. 2), suggesting the importance of this route in the introduction and spread of the virus into Uganda. Most of the genomes from truck drivers sampled at ports of entry (POEs) bordering Kenya belonged to lineage B.1 and B.1.393, which is consistent with the pattern reported in Kenya^10^. However, genomes identified from truck drivers from Tanzania and from the Elegu POE bordering South Sudan, albeit small numbers, belonged to both the A and B.1 lineages. Continued monitoring of truck drivers coming in and out of Uganda provides a useful description of the inland circulation of strains in this part of world, where genomic surveillance is not as detailed as in other parts of the world.

## Emergence of A.23 and A.23.1

**Fig. 3:**
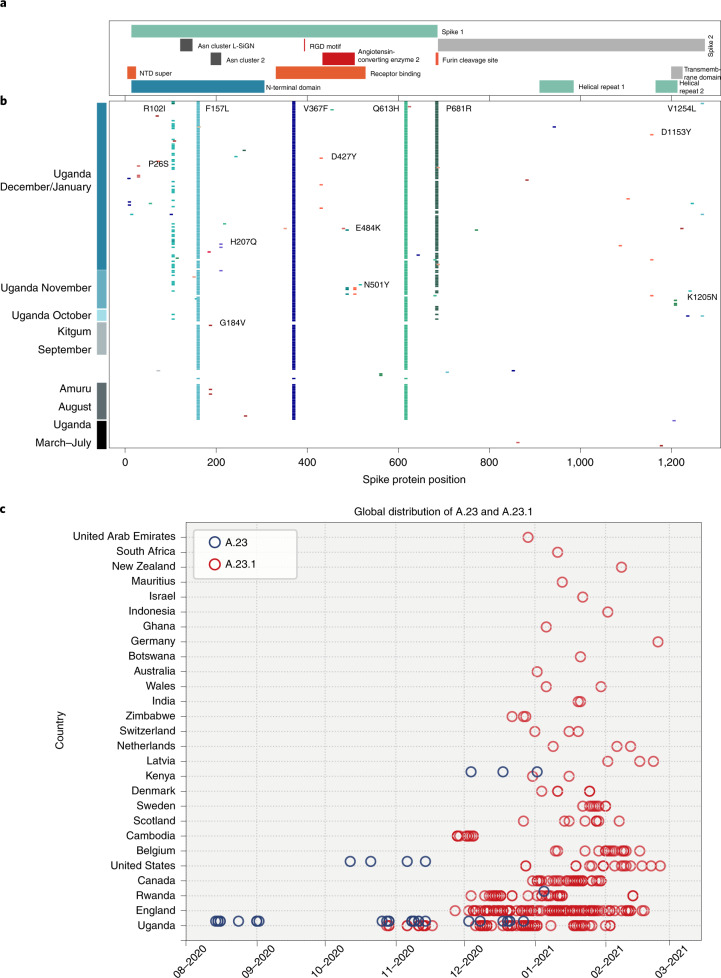
Spike protein changes in lineage A.23 and A.23.1 relative to the SARS-CoV-2 reference strain (NC_045512) encoded protein. **a**, The locations of important spike protein features are indicated. **b**, Each line represents the encoded spike protein sequence from a single genome, ordered by date of sample collection (bottom earliest, top most recent). Sequences from Amuru in August 2020, Kitgum in September 2020 and Uganda in October, November and December 2020/January 2021 are indicated. Coloured markers indicate the positions of amino acid substitutions from the reference strain sequence; only substitutions observed in multiple genomes are annotated with the annotation (original amino acid position, new amino acid) and the labels were placed as close as possible to the substitution. **c**, Current global temporal distribution of A.23 and A.23.1. All available SARS-CoV-2 genomes annotated as complete and lineage A from GISAID were retrieved on 4 February 2021 and lineage-typed using pangolin and confirmed as A.23 and A.23.1 by extracting and examining the encoded spike protein. All new Uganda A.23 and A.23.1 reported in this study were also included. Genomes were plotted by country and sample collection date. Source data

**Extended Data Fig. 1:**
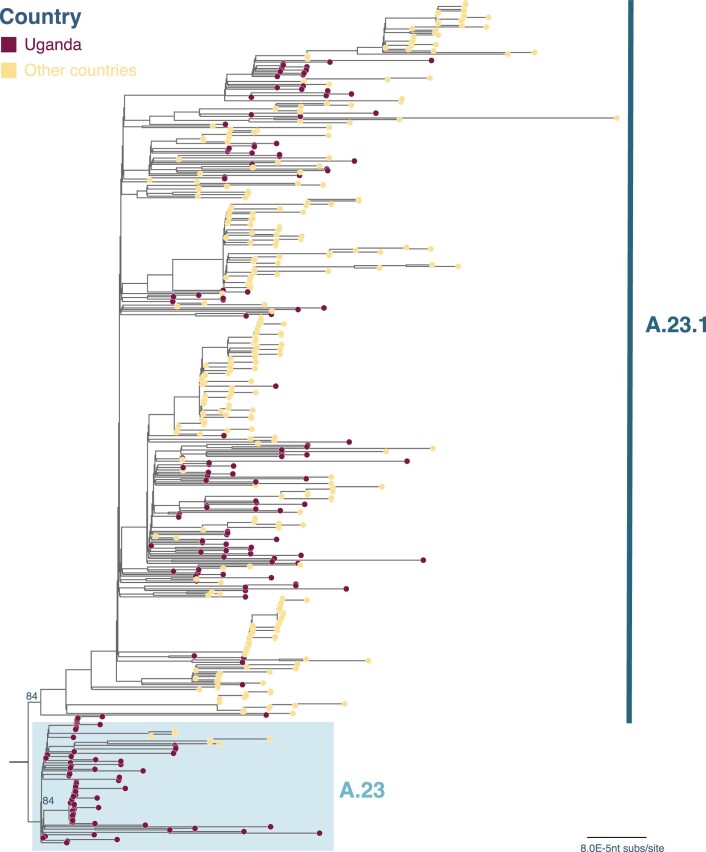
Maximum-likelihood phylogenetic tree comparing Uganda lineage A.23 and A.23.1 strains to global lineage A.23 and A.23.1 genomes. A maximum-likelihood (ML) phylogenetic tree comparing Ugandan A.23 and A.23.1 (n = 191) with the global A.23 and A.23.1 (N = 336). The tree was rooted by the A.23 lineage and strains were coloured according to the countries where they were identified. Branch length was drawn to the scale of number of nucleotide substitutions per site and only bootstrap values at the major nodes were shown. The tree was visualised in Figtree^3^. Source data

**Extended Data Fig. 2:**
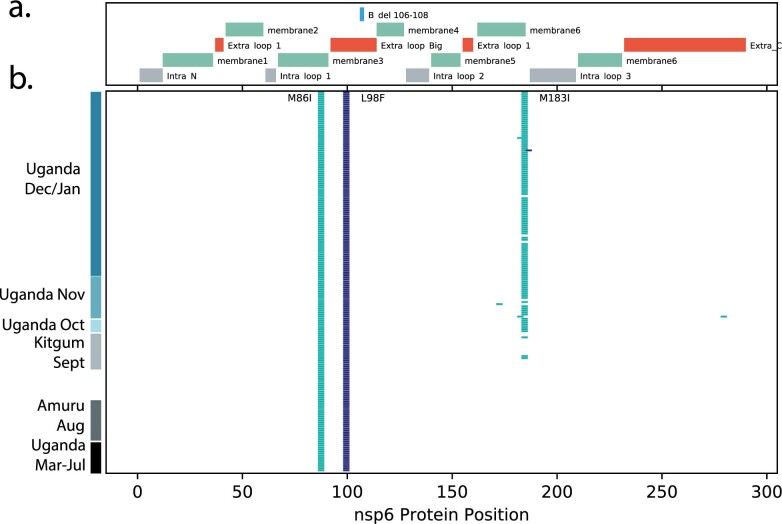
Changes in A.23/A.23.1 nsp6 protein. The encoded nsp6 protein from all Ugandan A.23 and A.23.1 genomes gather, aligned and compared to the nsp6 protein from GenBank NC_045512.2. Panel a: The locations of important nsp6 protein features are indicated based on the analysis of nsp6 from Benvenuto et al.^4^. Intra_N: intravesicular amino-terminal region, Extra_loop_1: extravesicular loop1, Intra_loop_1: intravesicular loop 1, B_del 106-108: the region of nsp6 deleted in the lineage B VOC genomes, Extra_loop_Big: large extravesicular loop, Intra_loop_2: intravesicular loop 2, Extra_loop_2: extravesicular loop 2, Intra_loop_3: intravesicular loop 3, Extra_C: carboxy-terminal extra-vesicular portion. All features with ‘membrane’ indicate membrane-spanning regions of nsp6. Panel b: Each line represents the encoded nsp6 protein sequence from a single genome, ordered by date of samples collection (bottom earliest, top most recent). Coloured markers indicate the positions of amino acid (aa) substitutions from the reference strain sequence, only substitutions observed in multiple genomes are annotated with the annotation (original aa position new aa) and the labels were placed as close as possible to the substitution. Source data

Outbreaks of SARS-CoV-2 infections were reported in the Amuru and Kitgum prisons in August 2020 (ref. ^13,14^). The SARS-CoV-2 genome sequences from individuals in the prisons were exclusively belonging to lineage A (Fig. 2) with three amino acid changes encoded in the spike protein (F157L, V367F and Q613H; Fig. 3) that now define lineage A.23. By October 2020, lineage A.23 viruses were also found outside of the prisons in a community sample from Lira (a town 140 km from Amuru), in two samples from the Kitgum hospital, in several community samples from Kampala, Jinja, Mulago, Tororo and Soroti as well as in 2 truck drivers collected at the POE bordering Kenya. By November 2020, the A.23 viruses had spread further to northern Uganda in Gulu and Adjumani, as observed in this study. Lineage A.23 viruses were not seen in Uganda (or anywhere in the world) before August 2020 (Fig. 3c), yet the A.23 viruses were attributed to 32% of the viruses in Uganda (Fig. 1) from June to August 2020 and 50% of the observed viruses in September–November 2020. In late October, the A.23.1, a variant evolving from A.23, with additional change in the spike protein (P681R) was observed (Fig. 3b,c); by December 2020 to January 2021, 90% of identified genomes (102 out of 113) belonged to the A.23.1 lineage (Figs. 1 and 2). The mutations in A.23.1 were consistent with evolution from an original A.23 virus observed in the Amuru/Kitgum cluster (Fig. 2 and Extended Data Fig. 1) as well as changes in nsp6 and ORF9 (Extended Data Figs. 2 and 4).

## Important changes observed in the spike protein

The spike protein is crucial for virus entry into host cells, for tropism and is a critical component of COVID-19 vaccine development and monitoring. The changes in spike protein observed in Uganda and the global A.23 and A.23.1 viruses are shown in Fig. 3b. Many amino acid changes were single events with no apparent transmission observed. However, the initial lineage A.23 genomes from Amuru and Kitgum encoded three amino acid changes in the exposed S1 domain of the spike protein (F157L, V367F and Q613H; Fig. 3b). The V367F change is reported to modestly increase infectivity^15^ and the Q613H change may have similar consequences as the D614G change observed in the B.1 lineage found predominantly in Europe and the US; in particular, D614G was reported to increase infectivity, spike trimer stability and furin cleavage^15–18^. These changes were not observed in previously reported genomes from Uganda^8^. Of some concern, the mutations E484K and N501Y amino acid changes in the receptor-binding domain were observed in the A.23 viruses identified in the Adjumani cases on 9–11 November 2020 (Fig. 3b). These two amino acid changes are shown to substantially compromise vaccine efficacy and antibody treatments.

**Fig. 4:**
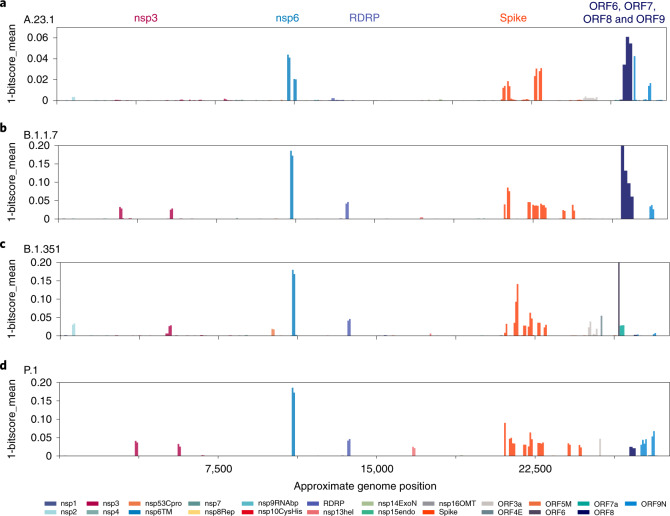
Protein changes across lineage variants. All forward open reading frames from the 35 early lineage B SARS-CoV-2 genomes were translated and processed into 44 amino acid peptides (with 22 amino acid overlap), clustered at 0.65 identity using uclust^39^, aligned with MAAFT^31^ and converted into pHMMs using HMMER-3 (ref. ^40^). The presence of each domain and its bit-score (a measure of the similarity between the query sequence and the sequences used for the pHMM^40^) was sought in each set of SARS-CoV-2 VOC genomes and the 1-mean of the normalized domain bit-scores was plotted across the genome (for example, 1—the similarity of the identified query domain to the reference lineage B SARS-CoV-2 domain). Domains are coloured by the proteins from which they were derived with the colour code is indicated below the figure. The genome positions of the indicated open reading frames are the following: nsp1: 250,805; nsp2: 806,2719; nsp3: 2720,8554; nsp4: 8555,10054; nsp53Cpro: 10055,10972; nsp6TM: 10973,11842; nsp7: 11843,12091; nsp8Rep: 12092,12685; nsp9RNAbp: 12686,13024; nsp10CysHis: 13025,13441; RDRP: 13442,16236; nsp13hel: 16237,18039; nsp14ExoN: 18040,19620; nsp15endo: 19621,20658; nsp16OMT: 20659,21552; spike: 21563,25384; ORF3a: 25393,26220; ORF4E: 26245,26472; ORF5M: 26523,27195; ORF6: 27202,27387; ORF7a: 27394,27759; ORF7b: 27756,27887; ORF8: 27894,28259; ORF9N: 28274,29533; ORF10: 29558,29712. Note that ORF7b and ORF10 are too small to be detected by this analysis method. **a**, The query set are 49 mostly Uganda lineage A.23.1 genomes. **b**, All B.1.1.7 full genomes lacking ambiguous nucleotides deposited in GISAID on 26 January 2021 are shown. **c**, All B.1.351 full genomes lacking ambiguous nucleotides present in GISAID on 26 January 2021 are shown. **d**, All P.1 full genomes lacking ambiguous nucleotides present in GISAID on 26 January 2021 are shown. Source data

Of concern, the recent Kampala and global A.23.1 virus sequences from December 2020 to January 2021 now encoded 4 or 5 amino acid changes in the spike protein (now defining lineage A.23.1) plus additional protein changes in nsp3, nsp6, ORF8 and ORF9 (Figs. 3b and 4). The substitution of proline by arginine at spike position 681 importantly adds a positively charged amino acid adjacent to the cleavage site for the host furin protease. An identical proline to arginine change enhances the fusion activity of the SARS-CoV-2 spike protein in in vitro experiments and this has been proposed to increase spike protein cleavage by the cellular furin protease^19^; importantly, a similar change (P681H) is encoded by the recently emerging VOC B.1.1.7 that is spreading globally across 75 countries as of 5 February 2021 (refs. ^5,20^). There are also changes in the spike N-terminal domain, a known target of immune selection, observed in samples from the Kampala A.23.1 lineage, including P26S and R102I (Fig. 3b). Additionally and importantly, an A.23.1 strain identified in Kampala on 11 December 2020 carried the E484K change in the receptor-binding domain, which may add further concern of this particular variant as it gains higher transmissibility and enhanced resistance to vaccines and therapeutics. Outside of the spike protein, a single nucleotide change (G27870T) leading to early termination of ORF7b (E39*) was observed in the A.23.1 from the community cases in Tororo in late December 2020. Although the clinical implication of this change is yet to be determined, it is important to document such changes for further follow-up.

## Lineage A designations

The viruses detected in Amuru and Kitgum met the criteria for a SARS-CoV-2 lineage^4,21^ by clustering together on a global phylogenetic tree, sharing epidemiological history and source from a single geographical origin and encoding multiple defining single-nucleotide polymorphism (SNPs). These features, especially the three spike changes F157L, Q613H and V367F, define the A.23 lineage. Continued circulation and evolution of A.23 in Uganda was observed and two additional changes in spike R102I and P681R were observed in December 2020 in Kampala, with the later amino acid change adding to the list of defining SNPs for the sublineage A.23.1 (F157L, V367F, Q613H and P681R). Additional changes in non-spike regions also define the A.23 and A.23.1 lineages, including: nsp3: E95K; nsp6: M86I, L98F; ORF 8: L84S, E92K; and ORF9 N: S202N, Q418H. These lineages can be assigned since pangolin v.2.1.10 and pangoLEARN data release 2021-02-01.

Screening SARS-CoV-2 genomic data from GISAID (12 March 2021), the A.23 and A.23.1 viruses were found in 26 countries outside of Uganda (Fig. 3c). A.23 was first observed in Uganda in August 2020, subsequently in the US in October and Kenya and Rwanda in December (Fig. 3c). The first A.23.1 genomes in Uganda were detected in community cases in Mbale on 28 October 2020 and in Jinja on 29 October 2020 and were soon spreading across the country in early November 2020. Outside Uganda, A.23.1 was found in England and Cambodia from the end of November and in Rwanda from the beginning of December. Of note, international flights out of Uganda were restarted on 1 October 2020 with flights to Europe, Asia and the US. Phylogenetic analysis supported the evolution of A.23 to A.23.1 (Extended Data Fig. 1).

## Additional changes in the A.23 and A.23.1 genomes from Uganda compared to other VOC genomes

Although a main focus has been on spike protein changes, there are changes in other genomic regions of the SARS-CoV-2 virus accompanying the adaptation to human infection. We employed profile Hidden Markov Models (pHMMs) prepared from 44 amino acid peptides across the SARS-CoV-2 proteome^22^ to detect and visualize protein changes from the early lineage B reference strain NC_045512. Measuring the identity score (bit-score) of each pHMM across a query genome provides a measure of protein changes in 44 amino acid steps across the viral genome (Fig. 4). This method applied to the A.23 and A.23.1 genome sequences revealed the changes in spike and changes in the transmembrane protein nsp6 and interferon modulators ORF8 and ORF9 (Fig. 4).

**Extended Data Fig. 3:**
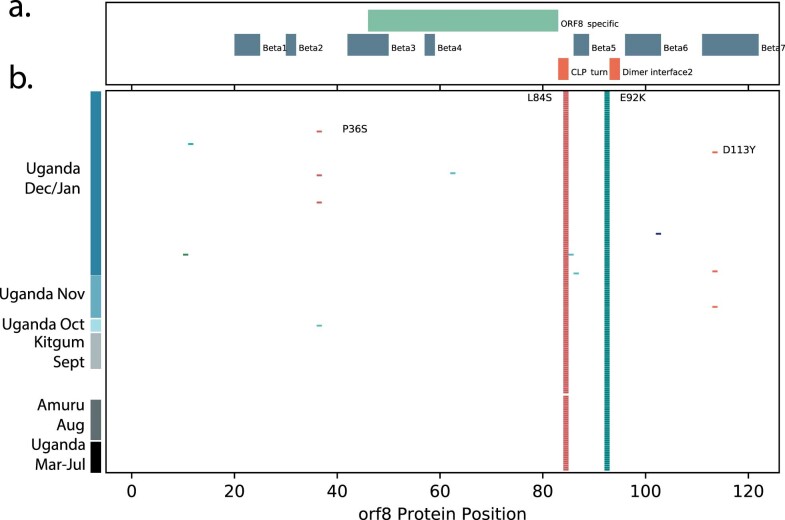
Changes in A.23/A.23.1 ORF8 protein. The encoded ORF8 protein from all Ugandan A.23 and A.23.1 genomes gather, aligned and compared to the ORF8 protein from GenBank NC_045512.2. Panel a: The locations of important ORF8 protein features are indicated based on the analysis of ORF8 from Flower et al.^5^. Features with ‘Beta’ indicate beta-sheets, ORF8_specific is a region unique to SARS-CoV-2 ORF8, CLP_turn: indicates a cysteine, Leucine, Proline motif essential for a fold in the mature protein, Dimer interface2 indicates the region of the protein the forms the interface between two monomers. Panel b: Each line represents the encoded ORF8 protein sequence from a single genome, ordered by date of samples collection (bottom earliest, top most recent). Coloured markers indicate the positions of amino acid (aa) substitutions from the reference strain sequence, only substitutions observed in multiple genomes are annotated with the annotation (original aa position new aa) and the labels were placed as close as possible to the substitution. Source data

**Extended Data Fig. 4:**
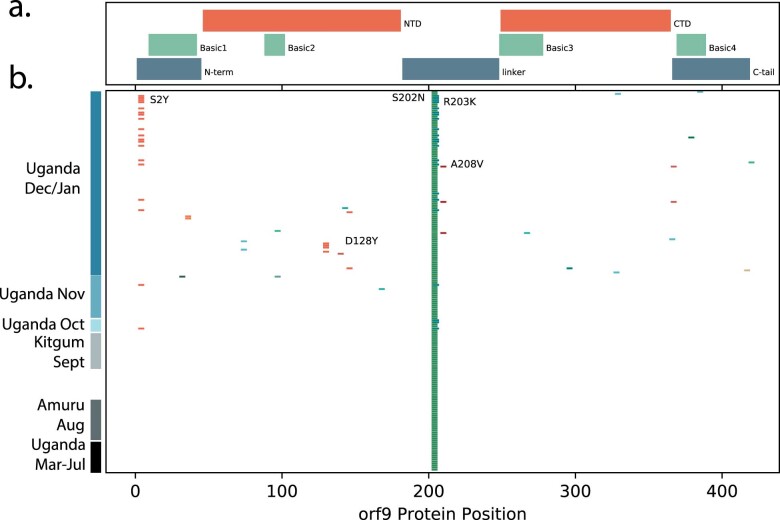
Changes in A.23/A.23.1 ORF9 protein. The encoded ORF9 protein from all Ugandan A.23 and A.23.1 genomes gather, aligned and compared to the ORF9 protein from GenBank NC_045512.2. Panel a: The locations of important ORF9 protein features are indicated based on the analysis of ORF9 from Chang et al.^6^. N-term: amino-terminal extension, NTD: amino-terminal domain, linker: linker region between the NTD and CTD, CTD: carboxy-terminal domain, C-tail: carboxy-terminal extension, Regions with ‘Basic’ indicate the 4 regions enriched in positively charged amino acids. Panel b: Each line represents the encoded ORF9 protein sequence from a single genome, ordered by date of samples collection (bottom earliest, top most recent). Coloured markers indicate the positions of amino acid (aa) substitutions from the reference strain sequence, only substitutions observed in multiple genomes are annotated with the annotation (original aa position new aa) and the labels were placed as close as possible to the substitution. Source data

We asked if a similar pattern of evolution was appearing in VOCs as SARS-CoV-2 adapted to human infection. We gathered the sets of genomes described in the initial published descriptions of these VOCs (B.1.1.7 (ref. ^5^), B.1.351 (ref. ^23^) or P.1 (ref. ^7^)) and applied the same pHMM analysis. Like A.23/A.23.1, the B.1.1.7 lineage encodes nsp6, spike, ORF8 and ORF9 changes as well as changes in nsp3 and RNA-dependent RNA polymerase (RDRP); Fig. 4b). Lineage B.1.351 encodes nsp3, nsp6, RDRP, spike and ORF6 changes (Fig. 4c) and lineage P.1 encodes nsp3, nsp6, RDRP, nsp13, spike and ORF8 and ORF9 changes (Fig. 4d). Although the exact amino acid and positions of change within the proteins differ in each lineage, there are some striking similarities in the common proteins that have been altered. Of interest, the nsp6 change present in B.1.1.7, B.1.351 and P.1 is a 3-amino acid deletion (106, 107 and 108) in a protein loop of nsp6 predicted to be on exterior of the autophagy vesicles on which the protein accumulates^24^. The three-amino acid nsp6 changes of lineage A.23.1 are L98F in the same exterior loop region; the M86l and M183I changes are predicted to be in intramembrane regions but adjacent to where the protein exits the membrane^24^ (Extended Data Fig. 2). The A23.1 *ORF8* gene encodes changes in the C-terminal domain (Extended Data Fig. 3). A compilation of the amino acid changes in A.23.1 and the VOC lineages is found in Supplementary Table 1 with proteins that are altered in all four lineages marked in red.

## Discussion

We report the emergence and spread of a SARS-CoV-2 variant of the A lineage (A.23.1) with multiple protein changes throughout the viral genome. The pattern of A.23.1 emergence and dominance has also been observed in the neighbouring country of Rwanda^25^. A similar phenomenon recently occurred with the B.1.1.7 lineage, detected first in the southeast of England^5^ and now globally, and with the B.1.351 lineage in South Africa^6^ and the P.1 lineage in Brazil^26^ suggesting that local evolution (perhaps to avoid the initial population immune responses) and spread may be a common feature of SARS-CoV-2. Importantly, lineage A.23.1 shares many features found in the lineage B VOCs, including alteration of key spike protein regions, especially the angiotensin-converting enzyme 2 binding region, which is exposed and immunogenic, the furin cleavage site and the 613/614 change that may increase spike multimer formation. The VOC and A.23.1 strains also encode changes in the similar region of the nsp6 protein, which may be important for altering cellular autophagy pathways that promote replication. Changes or disruption of ORF7, ORF8 and ORF9 are also present in the VOC and A.23.1. ORF8 changes or deletion probably indicates that this protein is unnecessary for human replication; similar deletions accompanied SARS-CoV-2 adaption to humans^27,28^.

**Extended Data Fig. 5:**
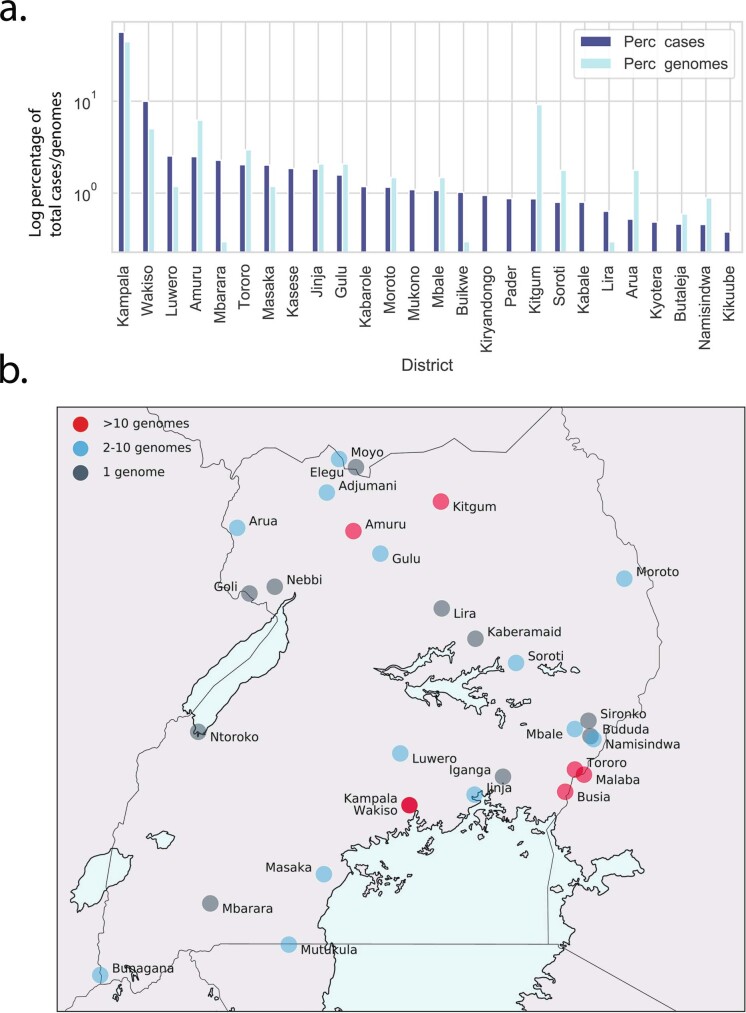
**a**, Percentage of total cases reported at the end of January 2021 were plotted by district (Perc_cases, dark blue bars). Only districts reporting 10 or more cases in the period were included. For the same districts, the percentage of total genomes obtained were plotted (Perc_genomes, light blue bars). The source data for Extended Data Fig. 5a can be found in Supplementary Table 2. **b**, District location of cases yielding full genome sequences. The district location in Uganda of cases from which full genome sequences are plotted on a map of Uganda. Districts with >10 genomes were marked in red, 2-10 genomes marked in blue and 1 genome marked in grey. Land masses are indicated in light grey, and lakes are indicate in pale blue. Source data

This study has potential limitations. We report the results of full-genome virus sequencing in a resource-limited region during a period with severe restraints on reagent procurement, travel and laboratory staffing; thus, total numbers were limited to 322 full genomes. Ideally, all positive cases in the country would be sequenced but this was practically not possible. On the other hand, the genome to case percentage we reported was 0.79% (322 genomes/40,490 cases), which is comparable with the case sequencing rate reported in South Africa (0.2%) and Nigeria (0.37%) for comparison. The geographical origin of the genomes (Extended Data Fig. 5) shows coverage across the country. Certainly, given the small number of genome sequences available from this study and from the region, we should caution that the particular evolutionary pathway proposed in this study (A.23 emergence in Uganda in August, evolution to A.23.1 and then spread to the region and globally) is supported by the available sequencing data but limited by the less then 100% sequence/case coverage and limited sampling in the region. Alternate pathways are possible if, for example, A.23.1 had evolved in Tanzania or another unsampled country and then moved into Uganda. Additionally, Uganda or the East Africa region does not have the resources to provide the detailed surveillance and diagnostic testing seen in Europe or North America, so national or sentinel surveillance may not be as detailed and comprehensive as that occurring in the north. Moreover, the MinION technology, like all other sequencing technologies currently in use (Illumina, Ion Torrent, Sanger Dideoxy), has a sequencing error profile. Nonetheless, MinION has been used to generate about 40% of over 1 million SARS-CoV-2 sequences now available in GISAID and is accepted as a reasonable sequencing technology. To limit any potential MinION sequencing errors in our sequences, we have reported and analysed only complete, high-coverage sequences (>10,000-fold coverage) and have manually checked all single nucleotide changes and deletions in the assembled genomes.

Independent of pangolin lineage assignation, it is clear that a SARS-CoV-2 lineage emerged (A.23) and evolved into a sublineage (A.23.1) that dominated the epidemic in Uganda by January 2021. This can be confirmed independently of pangolin use since we examined the maximum-likelihood phylogenetic trees (Fig. 2 and Extended Data Fig. 1) where the A.23 cluster of genomes is basal to the A.23.1 second cluster. Also independent of pangolin use, the pattern of amino acid changes observed with the substitutions observed in spike proteins from genomes identified as A.23 (F157L, V367F, Q613H) is a clear subset of the substitutions observed in the genomes designated A.23.1 (F157L, V367F, Q613H, P681R). Further support for the pangolin lineage assignation can be seen in the global timing of the observations of the two lineages illustrated in Fig. 3c, with the lineage A.23 cases observed before the A.23.1 samples. Certainly, the temporal pattern could have occurred by chance in a few places due to sequencing capacity and coverage. However, the global temporal pattern, particularly occurring in countries with massively extensive sequencing efforts like the UK and US, would indicate that the phenomenon is consistent with A.23.1 evolving from A.23 and consistent with the lineage classification by the pangolin tool.

We suspect that emerging SARS-CoV-2 lineages may be adjusting to infection and replication in humans and it is notable that the VOC and A.23.1 lineage share some common features in their evolution. The spike changes are best understood due to the massive global effort to define the receptor and develop vaccines against the infection. The analysis reported in Fig. 4 reveals common functions of SARS-CoV-2 that have been altered in all four variants, especially nsp6 and ORF8 and ORF9. The functional consequences of the additional non-spike changes warrant additional studies and the current analysis may focus the efforts of the proteins that are commonly changed in the variant lineages. Finally, determining the susceptibility of A.23.1 to vaccine immune responses is of great importance as vaccines become available in this part of Africa.

## Methods

### Statistics and reproducibility

No statistical method was used to predetermine sample size. The experiments were not randomized and the investigators were not blinded to allocation during the experiments and outcome assessment.

### Sample collection, whole-genome MinION sequencing and genome assembly

SARS-CoV-2 PCR with reverse transcription-positive samples were obtained from the Central Public Health Laboratories (Kampala, Uganda). All testing facilities across the country contribute to the sample collection at Central Public Health Laboratories and the sample catchment area is country-wide, including clinical sites, testing sites at border crossings and commercial laboratories testing the entry and exit of international travellers. The fraction of genomes per district compared to cases per district is shown in Extended Data Fig. 5a and the geographical source of the samples across Uganda is shown in Extended Data Fig. 5b. The samples reported in this manuscript span the period from the first positive case in Uganda (21 March 2020) until 23 January 2021. We attempted to sequence all samples that could be shared with us and each sample was only sequenced once (no replication was performed).

The nucleic acid extracted from samples was converted to complementary DNA and amplified using a SARS-CoV-specific 1,500-base pair amplicon spanning the entire genome as described previously^29^. The resulting DNA amplicons were used to prepare sequencing libraries, barcoded individually and then pooled to sequence on MinION R.9.4.1 flow cells, according to the manufacturer’s standard protocol.

Genome assemblies were performed as described previously^8^. Briefly, reads from FAST5 files were base-called and demultiplexed using Guppy v.3.6 running on the UMIC HPC. Adaptor and primer sequences were removed using Porechop v.0.2.4 (https://github.com/rrwick/Porechop) and the resulting reads were mapped to the reference genome Wuhan-1 (GenBank NC_045512.2) using minimap2-2.17 (r941)^30^ and consensus genomes were generated in Geneious Prime 2021.1.1 (Biomatters). Genome polishing was performed in Medaka v.1.3.4 and SNPs and mismatches were checked and resolved by consulting raw reads. To limit any possible MinION sequencing errors in our sequences, we have reported only high-coverage sequences and have manually checked all single nucleotide changes and deletions in the assembled genomes; non-supported changes have been replaced with NS.

### Phylogenetic analyses

For the local Uganda virus comparison, all available genomes from Uganda (*n* = 322) were aligned using MAFFT v.7.477 (ref. ^31^) and manually checked in AliView v.1.27 (ref. ^32^). The 5′ and 3′ untranslated regions were trimmed. The maximum-likelihood phylogenetic tree was constructed using RAxML-NG v.1.0.2 (ref. ^33^) under the GTR + I + G4 model as the best-fitted substitution model according to the Akaike information criterion determined by ModelTest-NG v.0.1.7 (ref. ^34^) and run for 100 pseudo-replicates. The resulting tree was visualized in FigTree v.1.4.4 (ref. ^35^) and rooted at the point of splitting lineages A and B^36–38^.

For the phylogenetic analyses of the Uganda lineage A.23 and A.23.1 strains comparing these to the global A.23/A.23.1 strains, the global SARS-CoV-2 lineage A.23 (*n* = 8) and A.23.1 (*n* = 38) genomes were retrieved from GISAID on 12 March 2021. These global A.23/A.23.1 genomes combined with the Ugandan A.23/A.23.1 genomes (*n* = 191) were aligned using MAFFT and manually checked in AliView; this was followed by trimming the 5′ and 3′ untranslated regions. The global and Ugandan A.23/A.23.1 genomes were used to construct a maximum-likelihood tree under the GTR + I + G4 model as the best-fitted substitution model according to the Akaike information criterion determined by ModelTest-NG^34^ and run for 100 pseudo-replicates using RAxML-NG. The resulting tree was visualized in FigTree and rooted using the A.23 lineage.

The pHMM domain analysis of A.23/A.23.1 and VOC genomes was performed as described previously^22^ with some changes. A database of pHMMs was generated from the first 65 lineage B SARS-CoV-2 genome sequences. All 3 forward open reading frames of each genome were translated computationally and then sliced into a 44-amino acid segment overlapping with 22 amino acids. All 44 amino acid query peptides were then clustered with the uclust module from usearch11.0.667_i86osx32 (ref. ^39^) and their original identity and coordinates determined by BLASTp search against a protein database made from the NC_045512 reference strain.

Query sets of genomes were processed to remove any genomes containing ambiguous nucleotides, which disrupt the HMM scoring process. The hmmscan function from HMMER v.3.3.2 (ref. ^40^) was used with the early B database. Query matches were identified using an E-value cut-off of 0.0001; the bit-score values for each hit (a measure of the distance between the query 44-amino acid peptide and the lineage B reference) was collected. Bit-scores for each domain were normalized by dividing each query score by the maximum score for that domain (x/x_max). In all analyses, the original lineage B NC_045512 reference genome was included to define the maximum bit-score.

### Reporting Summary

Further information on research design is available in the Nature Research Reporting Summary linked to this article.

## Supplementary information

Supplementary Information Supplementary Tables 1 and 2.

Reporting Summary

## Data Availability

All data, specialized code and instruction relevant to this manuscript are available at the GitHub repository https://github.com/mlcotten13/SARSCOV2_NatMicro. In addition, the SARS-CoV-2 genomes are available on GISAID (https://www.gisaid.org/) under accession nos. EPI_ISL_954226–EPI_ISL_954300, EPI_ISL_955136 and EPI_ISL_1469313–EPI_ISL_1469432. We have also deposited a complete alignment of these sequences in the Github repository. Source data are provided with this paper.

## Source data

Source Data Fig. 1 Source data for Fig. 1. Source Data Fig. 2 Multiple sequence alignment for Fig. 2. Source Data Fig. 3 Multiple sequence alignment for Fig. 3: CSV table with global A.23. and A.23.1 reports. Source Data Fig. 4 Source data for Fig. 4. Source Data Extended Data Fig. 1 Multiple sequence alignment for Extended Data Fig. 1. Source Data Extended Data Fig. 2 Multiple sequence alignment for Extended Data Fig. 2. Source Data Extended Data Fig. 3 Multiple sequence alignment for Extended Data Fig. 3. Source Data Extended Data Fig. 4 Multiple sequence alignment for Extended Data Fig. 4. Source Data Extended Data Fig. 5 Data tables for Extended Data Fig. 5a,b.

## References

[1] Holmes, E. C. & Zhang, Y.-Z. Novel 2019 coronavirus genome. *Virological.org* http://virological.org/t/319 (2020).

[2] Li Q (2020). Early transmission dynamics in Wuhan, China, of novel coronavirus-infected pneumonia. N. Engl. J. Med..

[3] Yang X (2020). Clinical course and outcomes of critically ill patients with SARS-CoV-2 pneumonia in Wuhan, China: a single-centered, retrospective, observational study. Lancet Respir. Med..

[4] Rambaut A (2020). A dynamic nomenclature proposal for SARS-CoV-2 lineages to assist genomic epidemiology. Nat. Microbiol..

[5] Volz, E. et al. Transmission of SARS-CoV-2 Lineage B.1.1.7 in England: insights from linking epidemiological and genetic data. Preprint at *medRxiv*10.1101/2020.12.30.20249034 (2021).

[6] Tegally, H. et al. Emergence and rapid spread of a new severe acute respiratory syndrome-related coronavirus 2 (SARS-CoV-2) lineage with multiple spike mutations in South Africa. Preprint at *medRxiv*10.1101/2020.12.21.20248640 (2020).

[7] Voloch, C. M. et al. Genomic characterization of a novel SARS-CoV-2 lineage from Rio de Janeiro, Brazil. *J. Virol*. 10.1128/JVI.00119-21 (2021). 10.1128/JVI.00119-21 PMC8139668 33649194

[8] Bugembe DL (2020). Main routes of entry and genomic diversity of SARS-CoV-2, Uganda. Emerg. Infect. Dis..

[9] Hadfield J (2018). Nextstrain: real-time tracking of pathogen evolution.. Bioinformatics.

[10] Githinji, G. et al. Tracking the introduction and spread of SARS-CoV-2 in coastal Kenya. Preprint at *medRxiv*10.1101/2020.10.05.20206730 (2020). 10.1038/s41467-021-25137-x PMC8355311 34376689

[11] Page, A. J. et al. Large scale sequencing of SARS-CoV-2 genomes from one region allows detailed epidemiology and enables local outbreak management. Preprint at *medRxiv*10.1101/2020.09.28.20201475 (2020). 10.1099/mgen.0.000589 PMC8461472 34184982

[12] da Silva Filipe A (2021). Genomic epidemiology reveals multiple introductions of SARS-CoV-2 from mainland Europe into Scotland. Nat. Microbiol..

[13] Amuru prison closed as 153 test positive for Covid-19. *Daily Monitor* https://www.monitor.co.ug/uganda/news/national/amuru-prison-closed-as-153-test-positive-for-covid-19-1924660 (2020).

[14] Nankunda, P. COVID-19: Uganda registers 318 new cases in a single day. *MSN News* https://www.msn.com/en-xl/news/other/covid-19-uganda-registers-318-new-cases-in-a-single-day/ar-BB18gprA (2020).

[15] Li Q (2020). The impact of mutations in SARS-CoV-2 spike on viral infectivity and antigenicity. Cell.

[16] Nguyen HT (2020). Spike glycoprotein and host cell determinants of SARS-CoV-2 entry and cytopathic effects. J. Virol..

[17] Gobeil SM-C (2021). *D614G* mutation alters SARS-CoV-2 spike conformation and enhances protease cleavage at the S1/S2 junction. Cell Rep..

[18] Volz E (2021). Evaluating the effects of SARS-CoV-2 spike mutation D614G on transmissibility and pathogenicity. Cell.

[19] Hoffmann M, Kleine-Weber H, Pöhlmann S (2020). A multibasic cleavage site in the spike protein of SARS-CoV-2 is essential for infection of human lung cells. Mol. Cell.

[20] O’Toole, Á. et al. *B.1.1.7 2021-02-05 Report* (2021); https://cov-lineages.org/global_report_B.1.1.7.html

[21] O’Toole, Á., Hill, V., McCrone, J. T., Scher, E. & Rambaut A. Pangolin COVID-19 Lineage Assigner (2020); https://pangolin.cog-uk.io/

[22] Phan MVT (2018). Identification and characterization of Coronaviridae genomes from Vietnamese bats and rats based on conserved protein domains. Virus Evol..

[23] Tegally H (2021). Emergence of a SARS-CoV-2 variant of concern with mutations in spike glycoprotein. Nature.

[24] Benvenuto D (2020). Evolutionary analysis of SARS-CoV-2: how mutation of Non-Structural Protein 6 (NSP6) could affect viral autophagy. J. Infect..

[25] Butera, Y. et al. Genomic sequencing of SARS-CoV-2 in Rwanda: evolution and regional dynamics. Preprint at *medRxiv*10.1101/2021.04.02.21254839 (2021).

[26] Voloch CM (2021). Genomic characterization of a novel SARS-CoV-2 lineage from Rio de Janeiro, Brazil. J. Virol..

[27] Su YCF (2020). Discovery and genomic characterization of a 382-nucleotide deletion in ORF7b and ORF8 during the early evolution of SARS-CoV-2. mBio.

[28] Chinese SARS Molecular Epidemiology Consortium. (2004). Molecular evolution of the SARS coronavirus during the course of the SARS epidemic in China. Science.

[29] Cotten M, Bugembe DL, Kaleebu P, Phan MVT (2021). Alternate primers for whole-genome SARS-CoV-2 sequencing. Virus Evol..

[30] Li H (2018). Minimap2: pairwise alignment for nucleotide sequences. Bioinformatics.

[31] Katoh K, Standley DM (2013). MAFFT multiple sequence alignment software version 7: improvements in performance and usability. Mol. Biol. Evol..

[32] Larsson A (2014). AliView: a fast and lightweight alignment viewer and editor for large datasets. Bioinformatics.

[33] Kozlov AM, Darriba D, Flouri T, Morel B, Stamatakis A (2019). RAxML-NG: a fast, scalable and user-friendly tool for maximum likelihood phylogenetic inference. Bioinformatics.

[34] Darriba D (2020). ModelTest-NG: a new and scalable tool for the selection of DNA and protein evolutionary models. Mol. Biol. Evol..

[35] Rambaut, A. FigTree (2019); http://tree.bio.ed.ac.uk/software/figtree

[36] Singer, J. B., Gifford, R., Cotten, M. & Robertson D. L. CoV-GLUE (2020); http://cov-glue.cvr.gla.ac.uk/

[37] Flower TG (2021). Structure of SARS-CoV-2 ORF8, a rapidly evolving immune evasion protein. Proc. Natl Acad. Sci. USA.

[38] Chang C, Hou M-H, Chang C-F, Hsiao C-D, Huang T (2014). The SARS coronavirus nucleocapsid protein—forms and functions. Antiviral Res..

[39] Edgar RC (2010). Search and clustering orders of magnitude faster than BLAST. Bioinformatics.

[40] Eddy SR (2011). Accelerated profile HMM searches. PLoS Comput. Biol..

[41] Bruls, M., Huizing, K. & van Wijk, J. J. in *Data Visualization 2000* (eds de Leeuw, W. C. & van Liere, R.) (Springer, 2000).

