# Supplementary material for: Emergence and spread of a SARS-CoV-2 lineage A variant (A.23.1) with altered spike protein in Uganda
Source: Nat Microbiol. 2021 Jun 23;6(8):1094–101. doi: 10.1038/s41564-021-00933-9 (PMC8318884; doi:10.1038/s41564-021-00933-9)
Supplement: Supplementary file 1 — Supplementary Tables 1 and 2. [file 41564_2021_933_MOESM1_ESM.pdf]

nature portfolio

https://doi.org/10.1038/s41564-021-00933-9

Supplementary information

Emergence and spread of a SARS-CoV-2 lineage A variant (A.23.1) with altered spike protein in Uganda

In the format provided by the authors and unedited

Supplementary Table 1. Summary of replacements in the A.23.1 and 3 VOC lineages[1].

Lineage	nsp2	nsp3	nsp5	nsp6	nsp12	spike	ORF3a	ORF4 E	ORF8	ORF9 N
A.23.1		nsp3: E95K		nsp6: M86I		S: R102I			ORF 8: L84S	N: S202N
A.23.1				nsp6: L98F		S: F157L			ORF 8: E92K	N: Q418H
A.23.1				nsp6: M183I		S: V367F				
A.23.1						S: Q613H				
A.23.1						S: P681R				
B.1.1.7		nsp3_T183I		nsp6_del_11288_11296	nsp12_P323L	S_N501Y			ORF 8_Q27*	N_D3L
B.1.1.7		nsp3_A890D				S_A570D			ORF 8_R52I	N_G204R
B.1.1.7		nsp3_I1412T				S_D614G			ORF 8_Y73C	N_R203K
B.1.1.7						S_P681H				N_S235F
B.1.1.7						S_T716I				
B.1.1.7						S_S982A				
B.1.1.7						S_D1118H				
B.1.1.7						S_del_21765_21770				
B.1.1.7						S_del_21991_21993				
B.1.351	nsp2_T85I	nsp3_K837N	nsp5_K90R	nsp6_del_11288_11296	nsp12_P323L	S_D80A	ORF 3a_Q57H	E_P71L		N_T205I
B.1.351						S_D215G				
B.1.351						S_K417N				
B.1.351						S_E484K				
B.1.351						S_N501Y				
B.1.351						S_D614G				
B.1.351						S_A701V				
B.1.351						S_non_cod_del_22281_22289				
B.1.281.1		nsp3_S370L		nsp6_del_11288_11296	nsp12_P323L	S_L18F	ORF 3a_S253P		ORF 8_E92K	N_P80R
B.1.281.1		nsp3_K977Q				S_T20N				N_R203K
B.1.281.1						S_P26S				N_G204R
B.1.281.1						S_D138Y				
B.1.281.1						S_R190S				
B.1.281.1						S_K417T				
B.1.281.1						S_E484K				
B.1.281.1						S_N501Y				
B.1.281.1						S_D614G				
B.1.281.1						S_H655Y				
B.1.281.1						S_T1027I				
B.1.281.1						S_V1176F				

1. Representative sets of each lineage (either all available gap-free full genomes (B.1.351 and B.1.281.1) or all genomes gap-free full genomes annotated as B.1.1.7 were analyzed using CoV-GLUE (2) to identify frequent amino acid replacements associated with each lineage.

Supplementary Table 2. Uganda SARS-CoV-2 cases and genome metrics[1].

District	Total_cases	Perc_cases	Genome_count	Perc_genomes
Kampala	16398	56.25578922	150	44.37869822
Wakiso	2907	9.97289787	17	5.029585799
Luwero	739	2.535249923	4	1.183431953
Amuru	728	2.497512779	21	6.213017751
Mbarara	664	2.277951216	1	0.295857988
Tororo	594	2.037805757	10	2.958579882
Masaka	589	2.02065251	4	1.183431953
Kasese	541	1.855981337	0	0
Jinja	533	1.828536142	7	2.071005917
Gulu	459	1.574668085	7	2.071005917
Kabarole	343	1.176712752	0	0
Moroto	338	1.159559505	5	1.479289941
Mukono	318	1.090946516	0	0
Mbale	312	1.07036262	5	1.479289941
Buikwe	298	1.022333528	1	0.295857988
Kiryandongo	276	0.94685924	0	0
Pader	254	0.871384953	0	0
Kitgum	253	0.867954304	31	9.171597633
Soroti	232	0.795910666	6	1.775147929
Kabale	232	0.795910666	0	0
Lira	185	0.634670143	1	0.295857988
Arua	152	0.521458712	6	1.775147929
Kyotera	141	0.483721568	0	0
Butaleja	134	0.459707023	2	0.591715976
Namisindwa	133	0.456276373	3	0.887573964
Kikuube	110	0.377371436	0	0

1. Percentage of total cases reported at the end of January 2021. Only districts reporting 10 or more cases in the period were included. For the same districts, the percentage of total genomes obtained are listed.

References

1. Áine O'Toole et al. Phylogenetic Assignment of Named Global Outbreak LINeages (PANGOLIN). 2020; Available from: https://github.com/cov-lineages/pangolin

2. Josh B. Singer, Gifford R, Cotten M, Robertson DL. CoV-GLUE project. 2020; Available from: http://cov-glue.cvr.gla.ac.uk/
