# Supplementary material for: Emergence and spread of a SARS-CoV-2 lineage A variant (A.23.1) with altered spike protein in Uganda
Source: Nat Microbiol. 2021 Jun 23;6(8):1094–101. doi: 10.1038/s41564-021-00933-9 (PMC8318884; doi:10.1038/s41564-021-00933-9)
Supplement: Supplementary file 2 — Reporting Summary [file 41564_2021_933_MOESM2_ESM.pdf]

nature research

Corresponding author(s): Matthew Cotten

Last updated by author(s)

Reporting Summary

Nature Research wishes to improve the reproducibility of the work that we publish. This form provides structure for consistency and transparency in reporting. For further information on Nature Research policies, see our Editorial Policies and the Editorial Policy Checklist.

Statistics

For all statistical analyses, confirm that the following items are present in the figure legend, table legend, main text, or Methods section.

n/a	Confirmed	
☒	☐	The exact sample size (n) for each experimental group/condition, given as a discrete number and unit of measurement
☒	☐	A statement on whether measurements were taken from distinct samples or whether the same sample was measured repeatedly
☒	☐	The statistical test(s) used AND whether they are one- or two-sided<br>*Only common tests should be described solely by name; describe more complex techniques in the Methods section.*
☒	☐	A description of all covariates tested
☒	☐	A description of any assumptions or corrections, such as tests of normality and adjustment for multiple comparisons
☒	☐	A full description of the statistical parameters including central tendency (e.g. means) or other basic estimates (e.g. regression coefficient) AND variation (e.g. standard deviation) or associated estimates of uncertainty (e.g. confidence intervals)
☒	☐	For null hypothesis testing, the test statistic (e.g. F, t, r) with confidence intervals, effect sizes, degrees of freedom and P value noted<br>*Give P values as exact values whenever suitable.*
☒	☐	For Bayesian analysis, information on the choice of priors and Markov chain Monte Carlo settings
☒	☐	For hierarchical and complex designs, identification of the appropriate level for tests and full reporting of outcomes
☒	☐	Estimates of effect sizes (e.g. Cohen's d, Pearson's r), indicating how they were calculated

Our web collection on statistics for biologists contains articles on many of the points above.

Software and code

Policy information about availability of computer code

Data collection	We use published software and programs to collect the data, as described in the manuscript.
Data analysis	We use published software and programs to analyse the data, as described in the manuscript.

For manuscripts utilizing custom algorithms or software that are central to the research but not yet described in published literature, software must be made available to editors and reviewers. We strongly encourage code deposition in a community repository (e.g. GitHub). See the Nature Research guidelines for submitting code & software for further information.

Data

Policy information about availability of data

All manuscripts must include a data availability statement. This statement should provide the following information, where applicable:

- Accession codes, unique identifiers, or web links for publicly available datasets
- A list of figures that have associated raw data
- A description of any restrictions on data availability

All genome sequences reported here are deposited in GISAID and available under accession numbers EPI_ISL_954226- -EPI_ISL_954300. A second tranche of genome sequences have been deposited and we are waiting for accession numbers

Field-specific reporting

Please select the one below that is the best fit for your research. If you are not sure, read the appropriate sections before making your selection.

☐ Life sciences ☐ Behavioural & social sciences ☒ Ecological, evolutionary & environmental sciences

For a reference copy of the document with all sections, see nature.com/documents/nr-reporting-summary-flat.pdf

Ecological, evolutionary & environmental sciences study design

All studies must disclose on these points even when the disclosure is negative.

Study description	We continually monitor SARS-CoV-2 as the virus enters and spreads in Uganda, by performing whole-genome deep sequencing of the virus.
Research sample	We used nucleic acid extracted from SARS-CoV-2 RT-PCR positive samples, which were obtained from Central Public Health Laboratory (Kampala, Uganda).
Sampling strategy	Samples were chosen to include random samples from each month with Ct-value below 30.
Data collection	We collected the date and location of sample collection and the Ct-value associated with the samples sequenced.
Timing and spatial scale	We collected the samples from the first SARS-CoV-2 case reported in Uganda on 21 March until early January 2021.
Data exclusions	*If no data were excluded from the analyses, state so OR if data were excluded, describe the exclusions and the rationale behind them, indicating whether exclusion criteria were pre-established.*
Reproducibility	A negative control is always included in every sequencing run to detect possible contamination or cross talk between samples.
Randomization	Samples were randomly chosen by our collaborators at Central Public Health Laboratory
Blinding	All samples were coded by unique lab identification, which anonymised all associated metadata. All we know are date of collection, location and Ct-values.

Did the study involve field work? ☐ Yes ☒ No

Reporting for specific materials, systems and methods

We require information from authors about some types of materials, experimental systems and methods used in many studies. Here, indicate whether each material, system or method listed is relevant to your study. If you are not sure if a list item applies to your research, read the appropriate section before selecting a response.

Materials & experimental systems

n/a	Involved in the study
☒	☐ Antibodies
☒	☐ Eukaryotic cell lines
☒	☐ Palaeontology and archaeology
☒	☐ Animals and other organisms
☒	☐ Human research participants
☒	☐ Clinical data
☒	☐ Dual use research of concern

Methods

n/a	Involved in the study
☒	☐ ChIP-seq
☒	☐ Flow cytometry
☒	☐ MRI-based neuroimaging
